# Systematic Review and Meta-Analysis Confirms Significant Contribution of Surfactant Protein D in Chronic Obstructive Pulmonary Disease

**DOI:** 10.3389/fgene.2019.00339

**Published:** 2019-04-17

**Authors:** Debparna Nandy, Nidhi Sharma, Sabyasachi Senapati

**Affiliations:** Department of Human Genetics and Molecular Medicine, Central University of Punjab, Bathinda, India

**Keywords:** SFTPD, COPD, AECOPD, rs721917, meta-analysis

## Abstract

**Background:** Surfactant protein D (SFTPD) is a lung specific protein which performs several key regulatory processes to maintain overall lung function. Several infectious and immune mediated diseases have been shown to be associated with SFTPD. Recent findings have suggested the serum concentration of SFTPD can be used as a diagnostic or prognostic marker for chronic obstructive pulmonary disease (COPD) and acute exacerbation COPD (AECOPD). But these findings lack replication studies from different ethnic populations and meta-analysis, to establish SFTPD as reliable diagnostic or prognostic biomarker for COPD and associated conditions.

**Methods:** We performed systematic literature search based on stringent inclusion and exclusion criteria to identify eligible studies to perform a meta-analysis. Our objective was to assess the predictability of serum SFTPD concentration and SFTPD allelic conformation at rs721917 (C > T) with COPD and AECOPD outcome. These variables were compared between COPD and healthy controls, where mean difference (MD), and odds ratio (OR) were calculated to predict the overall effect size. Review manager (RevMan-v5.3) software was used to analyse the data.

**Results:** A total of eight published reports were included in this study. Comparative serum SFTPD concentration data were extracted from six studies and three studies were evaluated for assessment of genetic marker from SFTPD. Our study identified strong association of elevated serum SFTPD with COPD and AECOPD. Significant association of risk was also observed for “T” allele or “TT” genotype of rs721917 from SFTPD with COPD and AECOPD.

**Conclusion:** Serum concentration and alleleic conformation of SFTPD has a significantly high predictive value for COPD and AECOPD. Thus, these can be tested further and could be applied as a predictive or prognostic marker.

## Introduction

Chronic Obstructive Pulmonary Disease (COPD) affects lungs and exhibits irreversible airflow conditions that leads to improper respiratory function (Carolan et al., 2014). COPD is a global disease burden which accounts for ~3 million deaths annually (Zemans et al., 2017) and is responsible for the increase in worldwide mortality and morbidity (Dickens et al., 2011). Chronic Obstructive Pulmonary Disease is projected to be the third leading cause of death by 2020 (Dickens et al., 2011). Chronic Obstructive Pulmonary Disease has multiple sub-phenotypic conditions like emphysema, lean body mass, mucus hypersecretion, and acute exacerbation (Dickens et al., 2011; Shakoori et al., 2012; Carolan et al., 2014). Each sub-phenotype is considered to be the outcome of different immune related pathways which are involved in COPD pathogenesis (Ishii et al., 2012).

Surfactant protein D (SF-D or SFTPD) is a highly lung specific glycoprotein secreted by type II alveolar cells and non-ciliated clara cells and functionally involved in maintaining the lung functions (Shakoori et al., 2012; Akiki et al., 2016). This multimeric glycoprotein belongs to lectin super family (Moreno et al., 2014) and takes part in immune regulation and maintenance of lung function (Ju et al., 2012). SFTPD is found to have three domains, namely: the collagen like domain, the neck domain, and the carbohydrate domain (Moreno et al., 2014). The carbohydrate binding domain is responsible for the maintenance of innate immune function in the lungs. Upon calcium binding, this calcium dependent protein cross-talks with defensin and other immunoregulatory molecules (Crouch and Wright, 2001; Jakel et al., 2013; Moreno et al., 2014). Due to considerably high molecular stability i.e., over 6 months in circulation, SFTPD has been investigated to establish it as a biomarker for pulmonary function (Holmskov et al., 2003; Hoegh et al., 2010).

Most COPD patients belong to the stable COPD category (SCOPD) followed by acute exacerbation COPD (AECOPD). AECOPD is characterized by sudden worsening of respiratory conditions including secretion of greenish phlegm (Shakoori et al., 2009). Trends of elevated serum SFTPD concentration among AECOPD patients compared to SCOPD or healthy control group have been reported. Elevated serum SFTPD among AECOPD patient group (*n* = 13; 227 ± 120 ng/ml), compared to SCOPD (*n* = 14; 151 ± 83 ng/ml), and control group (*n* = 54; 127 ± 65 ng/ml) was reported among Pakistanis (Shakoori et al., 2009). In another case-control study on a Chinese population, similar trend was observed, where serum SFTPD level was found to be significantly (*p* < 0.001) higher among AECOPD (*n* = 40; 235.22 ± 48.27 ng/ml) than SCOPD (*n* = 71; 153.54 ± 45.21 ng/ml) and control subjects (*n* = 60; 103.05 ± 24.97 ng/ml) (Ju et al., 2012). Serum SFTPD is often found to show association with different lung function parameters (Liu et al., 2014). Besides COPD, SFTPD is found to be associated with multiple pulmonary and other multifactorial diseases including lung cancer, interstitial pneumonia, asthma, viral infection, and other acute respiratory syndromes (Ishii et al., 2012; Carolan et al., 2014; Zemans et al., 2017). Serum SFTPD level can be pivotal in the diagnosis and monitoring of prognosis of various pulmonary conditions. Among COPD patients, serum concentration of SFTPD was found associated with BODE (body mass index, airflow obstruction, dyspnea, exercise capacity) index of severity (Ju et al., 2012) and mortality (Celli et al., 2012). However, its association with COPD severity was not observed in several other studies (Lomas et al., 2009; Liu et al., 2014; Akiki et al., 2016).

Genetic variations in *SFTPD* have also been established as informative genetic markers for COPD in different populations (Shakoori et al., 2012; Fakih et al., 2018). A non-synonymous variation rs721917:c.92T>C (p.Met31Thr) is associated with altered serum concentration of SFTPD and its multimerization (Sorensen et al., 2009). Presence of “T” or “C” alleles of rs721917 codes for methionine or threonine amino acids, respectively, at 31st position of SFTPD protein. Degradation of SFTPD from its multimerized (high molecular weight) to non-multimerized (low molecular weight) form is associated with respiratory diseases, including COPD (Fakih et al., 2018). This variation was also found associated with COPD among Mexicans and Europeans and with emphysema among Japanese populations (Guo et al., 2001; Foreman et al., 2011; Ishii et al., 2012; Horimasu et al., 2014). Other intronic or synonymous variations (rs2245121, rs911887, rs6413520, and rs7078012) were also identified as associated with altered serum concentration of SFTPD among Europeans (NETT-NAS and ECLIPSE cohorts) (Foreman et al., 2011).

In this study, we performed a systematic review and meta-analysis, with the objective to establish the potential of a single biomarker, SFTPD in identifying, and stratifying the different COPD sub-phenotype(s). We attempt to establish an association between variation in serum SFTPD concentration and the SFTPD genetic variation rs721917 with COPD and its sub- phenotype(s). The rationale of this study is to increase the power by considering multiple studies in a meta-analysis with similar environmental conditions, thereby evaluating the significance of SFTPD as an important diagnostic biomarker.

## Methodology

### Identification and Eligibility of Relevant Studies

**Figure 1:**
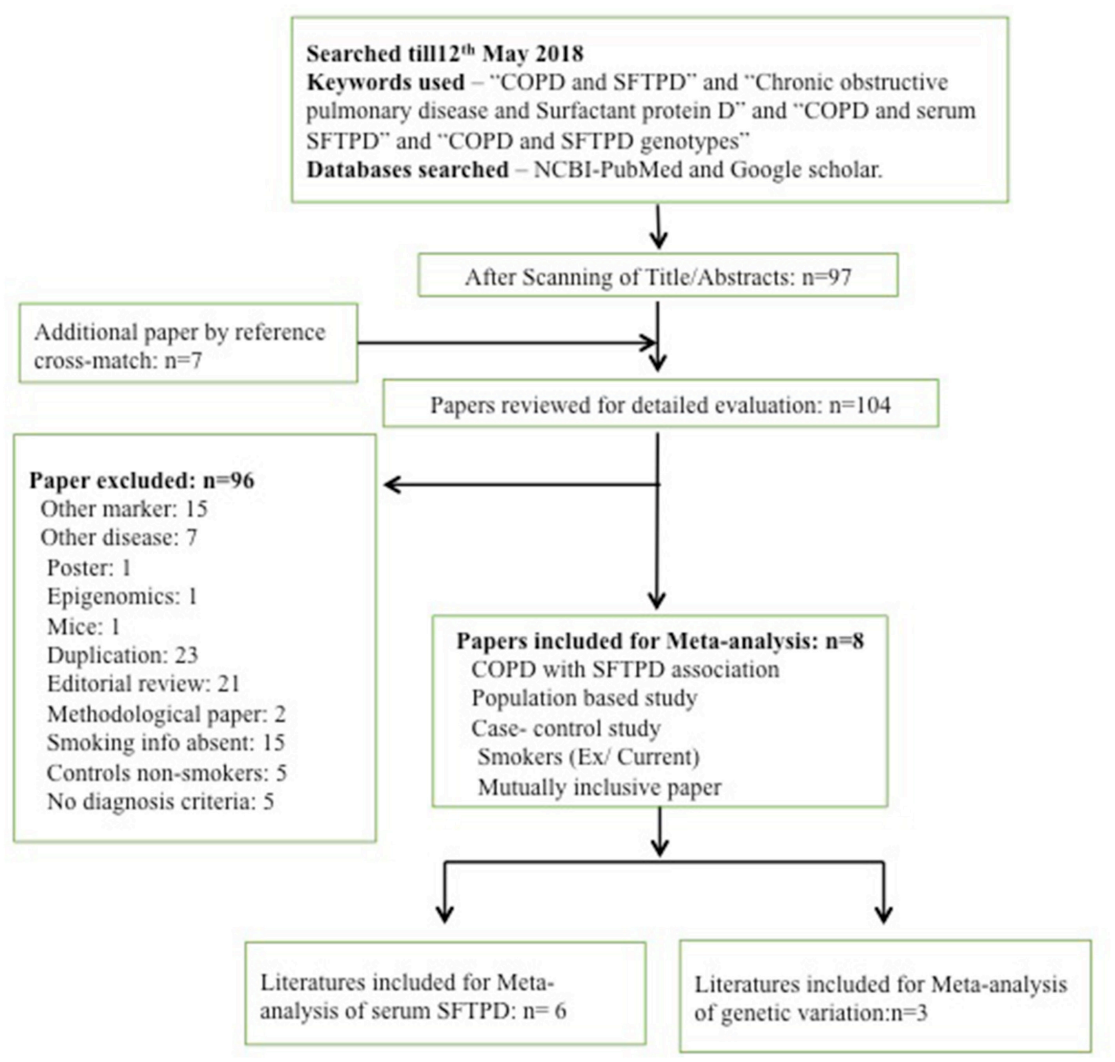
Flow–diagram for the literature search procedure performed for meta-analysis.

A search for eligible literature was done till May 12th, 2018. Databases used for the retrieval of eligible articles were PubMed (along with MESH database) and Google Scholar. The following keywords were used to retrieve all the publications: “COPD and SFTPD”; “Chronic obstructive pulmonary disease and surfactant protein D”; “COPD and serum SFTPD”; “COPD and SFTPD genotypes.” Publications with the desirable keywords were selected. Further publications were added from the cross-references of the retrieved articles. Details are given in the Figure 1.

### Study Inclusion/Exclusion Criteria

Scanning of publications with relevant titles and abstracts were done only for case-control studies encompassing COPD, and AECOPD as one of the major sub-phenotypes. No other sub-phenotypes of COPD were included in the study owing to maintain the focus area of the present meta-analysis. Only those studies were included where study participants were aged more than 35 years and all were smokers. We did not keep sex as a selection criteria. While for genetic polymorphisms, studies having information about rs721917 were only selected.

### Data Extraction

Data extraction from the eligible publications was done by two investigators independently and conflicts were resolved through group discussions. Following data was extracted from the finally selected publications:

- Protein biomarker: author names, number of participants, SFTPD serum/plasma level mean value (cases and controls), diagnostic criteria.
- Genetic biomarker (rs721917): author names, number of participants, allele distribution among cases and controls, population, and diagnostic criteria.

### Statistical Analysis

For statistical analysis, Review Manager (RevMan-v5.3) Copenhagen: The Nordic Cochrane Center, The Cochrane Collaboration, 2014, software was used. As serum biomarker level is a continuous variable mean difference (MD) was calculated. For genetic marker odds ratio (OR) for pooled data was calculated. Different genetic models such as, allelic model, dominant model, recessive model, and additive model were used to analyze the association. Heterogeneity among studies was calculated using *I*^2^ and chi^2^ tests, where *I*^2^ more than 50% and chi^2^ *p*-value <0.05 was considered significant heterogeneity. Both the analyses were done using fixed effect model. Meta-OR or Meta-MD were calculated using *Z*-test with 5% level of significance and 95% confidence interval. Possible publication bias was evaluated through visual inspection of funnel plots generated using the same software.

## Results

### Characteristics of Eligible Studies

Following online literature search, a total of 97 publications were obtained. Additionally seven publications were found through cross-references. However, based on our study inclusion-exclusion criteria, a total of 96 publications were excluded. Therefore, only eight publications were found eligible and taken forward for the meta-analysis (Figure 1). Eligible studies were reported between 2009 and 2017 (Supplementary Tables 1, 2). Out of these 96 publications, six were assessed to evaluate the risk of SFTPD serum concentration and three were assessed to evaluated for risk of genetic variation in *SFTPD* (rs721917) with overall COPD and acute exacerbation with COPD (AECOPD). Detail characteristics of these studies are presented in the Supplementary Tables 1, 2. No significant publication bias was observed among the studies included in this meta-analysis (Supplementary Figure 1).

### Association of Serum SFTPD With COPD

**Figure 2:**
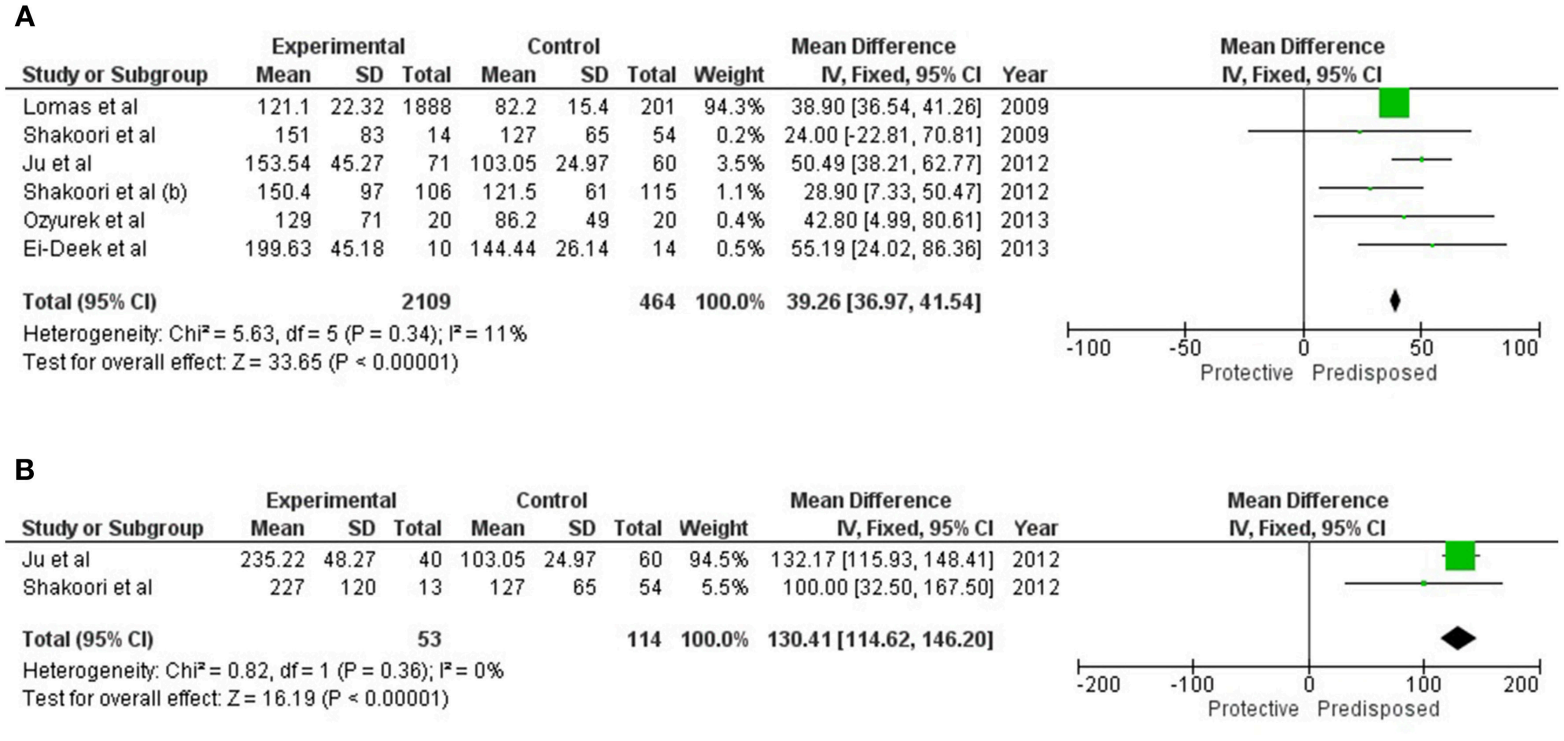
Forest plots showing results of meta-analysis to assess the association of serum SFTPD concentration with **(A)** Overall COPD, and **(B)** AECOPD. The Mean Difference between the COPD subjects and controls were used for estimating the variation among the same. Fixed model was used for meta-analysis as there were negligible study heterogeneity (*I*^2^ < 50%), in both the analyses.

Serum concentration of SFTPD (mean and SD) was available for a total of 2,109 cases and 464 healthy controls reported in eligible studies. Elevated serum SFTPD values were found to be significantly associated [M.D = 39.26 (36.97, 41.54; p_*Z* < 0.00001] with overall COPD (Table 1 and Figure 2A). Two of these studies were further assessed for evaluating the contribution of serum SFTPD level with AECOPD. Meta-analysis was performed on 53 cases and 114 controls. Elevated level of serum SFTPD was found associated with AECOPD [MD = 130.41(114.62, 46.20); p_*Z* < 0.00001] (Table 1 and Figure 2B).

**Table 1:** Summarized results for association of serum SFTPD concentration with COPD and AECOPD.

**Sr.no**	**Study no**.	**Stratification**	**COPD/Control**	**MD (95 % CI)**	**Effect size (Z)**	**P (Z test)**	**I^**2**^ (%)**	**Pheterogeneity**
1.	6	Overall COPD	2109/464	39.26 [36.97, 41.54]	33.65	<0.0001	11	0.34
2.	2	Acute exacerbation COPD (AECOPD)	53/114	130.41[114.62, 46.20]	16.19	<0.00001	0	0.36

### Association of *SFTPD* Genotype With COPD

**Figure 3:**
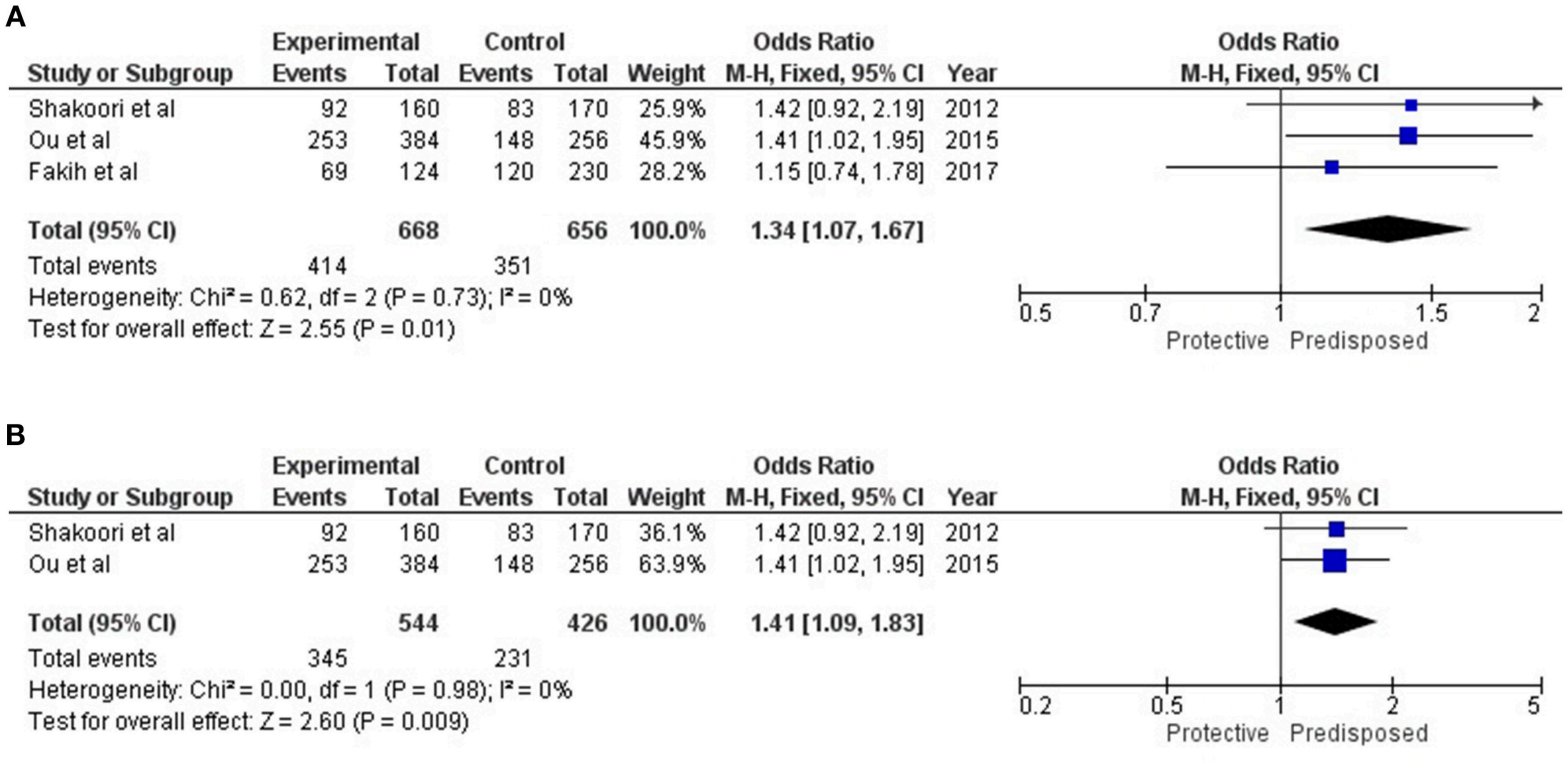
Forest plots showing results of meta-analysis to assess the association of SFTPD alleles (rs721917-T) with **(A)** Overall COPD, and **(B)** AECOPD; where meta-Odds Ratio was calculated based on the previously reported data. Fixed model statistics was used for meta-analysis as no study heterogeneity (*I*^2^ = 0) was observed.

All of these three reports on the association of SFTPD genetic variations with overall COPD were carried out on Asian populations. Cases in Chinese and Lebanese populations were diagnosed according to both American Thoracic Society (ATS) and GOLD Criteria, while the Pakistani population was diagnosed solely on the basis of GOLD criteria (Supplementary Table 2). For rs721917; allelic and genotypic (dominant and recessive) associations are summarized in Table 2. Under the allelic model of association, “T” allele was identified to confer risk for both COPD [OR = 1.34 (1.07–1.67); p_*Z* = 0.01] and AECOPD [OR = 1.41 (1.09–1.83); p_*Z* = 0.009] (Figure 3). Similarly, dominant model identified association of “TT” genotype with both COPD [OR = 1.41 (1.00–1.99); p_*Z* = 0.05] and AECOPD [OR = 1.50 (1.01–2.23); p_Z = 0.04] with marginal significance. Recessive model confirmed the protective role of “CC” genotype for both COPD [OR = 0.60 (0.39–0.94); p_*Z* = 0.02] and AECOPD [OR = 0.55 (0.33–0.92); p_*Z* = 0.02]. Dominant role and risk confers by the “T” allele was further confirmed by additive models of association (Table 2).

**Table 2:** Results of meta-analysis for alleles and genotypes of rs721917 under different genetic models.

**Sr. no**.	**Genetic model**	**Stratification**	**Study numbers**	**Cases/Control**	**OR (95 % CI)**	**Z_p**	***I*^**2**^ (%)**	**Heterogeneity *P*-value**
1.	Allelic model (T vs. C)	COPD	3	668/656	1.34 [1.07,1.67]	0.01	0	0.73
		AECOPD	2	544/426	1.41 [1.09, 1.83]	0.009	0	0.98
2.	Dominant model (TT vs. CT/CC)	COPD	3	334/328	1.41 [1.00, 1.99]	0.05	0	0.72
		AECOPD	2	272/213	1.50 [1.01, 2.23]	0.04	0	0.58
3.	Recessive model (CC vs. CT/TT)	COPD	3	334/328	0.60 [0.39, 0.94]	0.02	22	0.28
		AECOPD	2	272/213	0.55 [0.33, 0.92]	0.02	54	0.14
4a.	Additive model Homozygote comparison (TT vs. CC)	COPD AECOPD	3 2	162/150 135/99	1.88 [1.15, 3.09] 2.10 [1.18, 3.75]	0.01 0.01	0 0	0.49 0.34
4b.	Additive model Heterozygote comparison (TT vs. CT)	COPD AECOPD	3 2	293/264 241/172	1.30 [0.90, 1.86] 1.36 [0.90, 2.06]	0.16 0.14	0 0	0.62 0.14
4c.	Additive model Heterozygote comparison (CT vs. CC)	COPD AECOPD	3 2	213/140 168/153	1.51 [0.96, 2.38] 1.63 [0.95, 2.78]	0.08 0.08	37 66	0.20 0.08

*All analyses were done using Fixed effect model*.

## Discussion

Surfactant protein-D is a key innate immunity molecule with significant role in host defense. It has been reported as associated with several health conditions including COPD as well as various associated manifestations, such as AECOPD (Hartl and Griese, 2006). Recent genome-wide association studies had identified SFTPD as one of the most significant and well-replicated gene associated with COPD and it's allied complications (Kim et al., 2012; Hobbs et al., 2017). Significant difference in SFTPD serum concentration among the COPD and healthy controls can be used as criteria for disease diagnosis and/or prognosis. So far, except for emphysema (alpha-1 antitrypsin) no other biomarker is available for the diagnosis or evaluation the COPD prognosis and associated lung function. COPD and AECOPD are reported to be associated with elevated serum concentration of SFTPD (Lomas et al., 2009; Shakoori et al., 2009, 2012; Ju et al., 2012; El-Deek et al., 2013; Ozyurek et al., 2013). In contrary emphysema patients are found to have lower level of serum SFTPD than healthy people (Ishii et al., 2012).

This study is the first attempt to review and meta-analyze the existing published literature to assess the predictive value of SFTPD serum concentration or genotypes for COPD and AECOPD. Due to stringent study inclusion and exclusion criteria limited articles were found eligible for this study. Present study identified strong associations of elevated serum SFTPD level with both COPD and AECOPD. Extracted data from all the eligible studies were homogenous and no study selection bias was observed. As expected, serum SFTPD was observed more significantly associated with AECOPD (*p* < 0.00001; MD = 130.41) compared to COPD (*p* < 0.0001; MD = 39.26) when compared to healthy controls. However, as this study was performed on reported case-control based cross-sectional studies, causal effect relationship between the serum SFTPD level and COPD could not be affirmed. Recent evidences confirmed that elevated serum SFTPD can be used as a prognostic marker for COPD, as its serum concentration has been found significantly elevated among AECOPD compared to COPD (Shakoori et al., 2012; Ou et al., 2015).

Present study suggests the use of SFTPD as a biomarker to evaluate COPD. Since different range of SFTPD concentrations are found for different COPD and AECOPD conditions, single biomarker can be used for the diagnosis of COPD, and it's prognosis. Range of scale (SFTPD serum concentration) can be made to access the diagnosis and prognosis.

Limitations of this study include, less population numbers due to stringent inclusion and exclusion criteria. Population as a whole has been considered and not further stratified on their ethnicities. Studies with larger cohorts need to be conducted to confirm the association of serum SFTPD and its allelic conformation with COPD and AECOPD. Furthermore, to generalize these findings large-scale population based replication studies are warranted.

## Author Contributions

SS conceptualized the study. DN and NS performed the systematic review and meta-analysis. SS, DN, and NS wrote the manuscript. All authors reviewed and finalized the manuscript for submission.

### Conflict of Interest Statement

The authors declare that the research was conducted in the absence of any commercial or financial relationships that could be construed as a potential conflict of interest.
