# Supplementary material for: Systematic Review and Meta-Analysis Confirms Significant Contribution of Surfactant Protein D in Chronic Obstructive Pulmonary Disease
Source: Front Genet. 2019 Apr 17;10:339. doi: 10.3389/fgene.2019.00339 (PMC6479180; doi:10.3389/fgene.2019.00339)
Supplement: Supplementary file 1 [file Table_1.docx]

**Supplementary Tables**

| **Author name** | **Subjects** | **Mean Serum SFTPD concentration (ng/ml)** | | **Characteristics of COPD patients** |
| --- | --- | --- | --- | --- |
|  |  | **COPD** | **Healthy controls** |  |
| Shakoori *et al*., 2009 | 81 | 151.0 ± 83 | 127.0 ± 65 | Stable and Acute exacerbation. |
| Lomas *et al*., 2009 | 2385 | 121.1 ± 22.32 | 82.2 ± 15.4 | Age (40-75 yrs), Smokers (>10 pack years, GOLD (stage II, III, IV) |
| Ju *et al*., 2012 | 171 | 153.54 ± 45.21 | 103.05 ± 24.97 | Stable and Acute exacerbation, GOLD criteria |
| El Deek *et al*., 2013 | 90 | 199.63 ± 45.18 | 144.44 ± 26.14 | GOLD criteria, Smoker (non/current) |
| Ozyurek *et al*., 2013 | 60 | 129.0 ± 71 | 86.2 ± 49 | Age (>40 yrs), Smokers (>10 pack years), GOLD (stage I, II, III, IV) |
| Shakoori *et al*., 2012 | 221 | 150.4 ± 97 | 121.5 ± 61 | Lung function test by GOLD criteria, smokers (non/current/ex)  Exacerbation was diagnosed by history of dyspnoea, sputum volume, change in sputum color.  Pakistani men, age range 26-74 yrs. |

**Supplementary table 1**: Characteristics of studies included for the meta-analysis between serum SFTPD concentration and COPD/AECOPD.

**Supplementary table 2:** Characteristics of studies included for the meta-analysis between SFTPD genetic marker (rs721917, C > T) and COPD/AECOPD.

| **Author** | **Country** | **Ethnicity** | **Subjects** | **COPD Characteristics** |
| --- | --- | --- | --- | --- |
| Shakoori *et al*., 2012 | Pakistan | Asian | 80 COPD  85 Controls | COPD patients were diagnosed based on the FEV1/FVC values, as per GOLD criteria.  All study subjects were men.  Limited study power |
| Ou *et al*., 2015 | China | Asian | 192 COPD  128 Controls | COPD patients were diagnosed based on the FEV1/FVC values, as per GOLD and American Thoracic Society criteria. All were clinically stable.  All study subjects were men  Age > 40 yrs  Limited study power |
| Fakih *et al*.,  2017 | Lebanon | Asian | 62 COPD  115 Controls | Patients diagnosed according to GOLD criteria and American Thoracic Society |
