# Supplementary material for: Systematic Review and Meta-Analysis Confirms Significant Contribution of Surfactant Protein D in Chronic Obstructive Pulmonary Disease
Source: Front Genet. 2019 Apr 17;10:339. doi: 10.3389/fgene.2019.00339 (PMC6479180; doi:10.3389/fgene.2019.00339)
Supplement: Supplementary file 2 [file Image_1.pdf]

SUPPLEMENTARY FIGURES

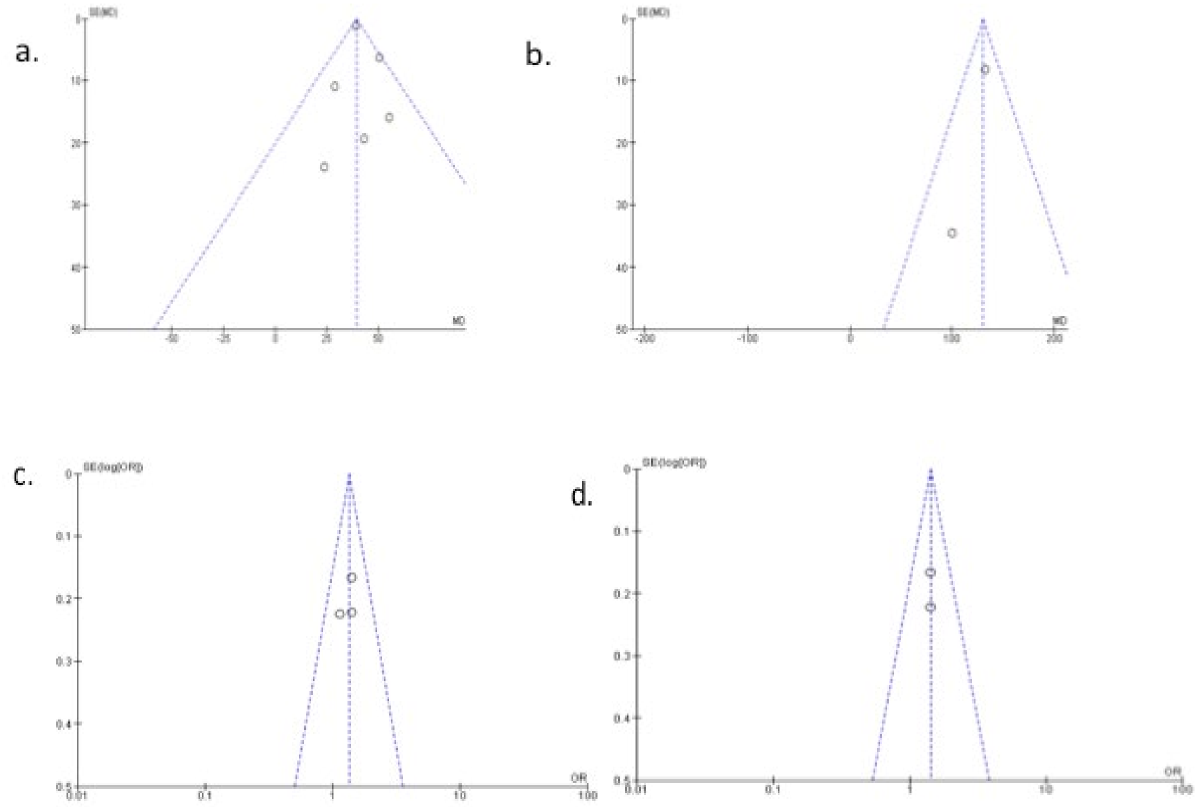

Supplementary Figure 1: Funnel plots were constructed to evaluate the publication bias in the study. (a.) Serum SFTPD level in overall COPD; (b.) Serum SFTPD level in AECOPD; (c.) rs721917 allelic association with overall COPD; (d.) rs721917 allelic association with AECOPD.
